# Enhanced IFN-γ, but not IL-2, response to *Mycobacterium tuberculosis* antigens in HIV/latent TB co-infected patients on long-term HAART

**DOI:** 10.1186/s12865-019-0317-9

**Published:** 2019-10-11

**Authors:** Girmay Desalegn, Aster Tsegaye, Dawit Gebreegziabiher, Abraham Aseffa, Rawleigh Howe

**Affiliations:** 1 Armauer Hansen Research Institute, Addis Ababa, Ethiopia; 2 Department of Medical Laboratory Sciences, Addis Ababa University, Addis Ababa, Ethiopia; 3 Department of Medical Microbiology and Immunology, Mekelle University, Mekelle, Ethiopia

**Keywords:** HIV, HAART, Latent TB infection, *M. Tuberculosis*, IFN-γ, IL-2, CD4^+^ T cells

## Abstract

**Background:**

HIV-infected individuals with latent TB infection are at increased risk of developing active TB. HAART greatly reduces the incidence rate of TB in HIV-infected patients and reconstitutes *Mycobacterium tuberculosis (M. tuberculosis)-*specific immune response in the first 12 months of therapy. The durability of the anti-mycobacterial immune restoration after a year of HAART however remains less investigated.

**Method:**

A cross-sectional study was conducted to evaluate *M. tuberculosis*-specific functional immune responses in HIV/latent TB co-infected patients who were on HAART for at least 1.5 up to 9 years as compared to HAART-naïve patients. Three-hundred sixteen HIV-infected patients without active TB were screened by tuberculin skin testing for *M. tuberculosis* infection and peripheral blood mononuclear cells (PBMCs) were isolated from 61 HIV/latent TB co-infected patients (30 HAART-naïve and 31 HAART-treated). IFN-γ and IL-2 ELISPOT as well as CFSE cell proliferation assays were performed after stimulation with *M. tuberculosis* antigens PPD and ESAT-6.

**Result:**

The median frequency of PPD and ESAT-6 specific IFN-γ secreting cells was significantly higher in the HAART-treated patients as compared to HAART-naïve patients, *p* = 0.0021 and *p* = 0.0081 respectively. However, there was no significant difference in the median frequency of IL-2 secreting cells responding to PPD (*p* = 0.5981) and ESAT-6 (*p* = 0.3943) antigens between HAART-naïve and-treated groups. Both IFN-γ and IL-2 responses were independent of CD4^+^ T cell count regardless of the HAART status. Notably, the frequency of PPD and ESAT-6 specific IL-2 secreting cells was positively associated with CD4^+^ T cell proliferation while inversely correlated with duration of HAART, raising the possibility that *M. tuberculosis*-specific IL-2 response that promote the antigen-specific CD4^+^ T cell proliferation diminish with time on antiretroviral therapy in HIV/latent TB co-infected patients.

**Conclusion:**

This study shows an increased *M. tuberculosis*-specific IFN-γ, but not IL-2, response in HIV/latent TB co-infected patients with long-term HAART, consistent with only partial immune restoration. Future studies should, therefore, be done to prospectively define the rate and extent to which functional immune responses to *M. tuberculosis* are restored after long-term HAART.

## Background

Tuberculosis (TB) is the one of the leading deadly diseases worldwide with over 90% of deaths occurring in developing countries [1]. TB is caused by direct exposure to *Mycobacterium tuberculosis (M. tuberculosis),* and/or reactivation of latent TB infection. Once infected with *M. tuberculosis,* only about 5–10% of people directly develop active TB while 90–95% remain latently infected [2, 3]. In 2014, approximately 1.7 billion people were latently infected with *M. tuberculosis* globally, low-and middle-income countries accounting for around 80% of the prevalence [4]. Immunocompetent individuals control the infection by containing the mycobacteria in an inactive or latent state. Both the innate and adaptive arms of the immune system are involved in a collaborative way to control infection with *M. tuberculosis* and subsequent disease. Various T cells produce potent cytokines and the interaction of these cells with infected macrophages are crucial for anti-mycobacterial protective responses [2, 3, 5–7]. People with latent TB infection have only 5–10% lifetime risk of reactivation [8]. However, following acquisition of HIV infection, the risk of reactivation of latent TB infection to active TB increases to 5–10% each year [3, 9]. This high rate of active TB development might be directly related to HIV-derived weakened host cell-mediated immunity in general, and impaired *M. tuberculosis*-specific immune responses in particular. *M. tuberculosis*-specific production of interferon-(IFN)-γ, interleukin (IL)-2 and Tumor necrosis factor (TNF)-α by T cells contribute substantially to elicit effective immunity to prevent reactivation of latent TB infection [7, 10–12].

Highly active anti-retroviral therapy (HAART) reduces the incidence rate of TB in people living with HIV. A meta-analysis of various studies has shown that HAART is associated with a 67% reduction (range: 61–73%) in TB incidence [13–16]. Reduction of TB incidence rate after initiation of HAART could likely be due to continuous CD4^+^ T cell recovery [14, 17] accompanied by restoration of functional anti-mycobacterial immunity. This is supported by studies which demonstrated initiation of HAART leads to rapid functional recovery of mycobacteria-specific immune responses, with increasing lymphocyte proliferation and secretion of IFN-γ by peripheral blood mononuclear cells (PBMCs) stimulated ex vivo with mycobacterial antigens [18–20]. However, most investigators have evaluated responses within 1 year of HAART initiation. Immune reconstitution occurs in two or more phases over time. CD4^+^ T cells recovery observed in the first year of HAART is rapid and prominent, and primarily a result of an immediate redistribution of memory CD4^+^ T-cells from the lymphoid tissues into the blood streams [21–23]. After a year of therapy, this phase is followed by a slow recovery of CD4^+^ T-cells and is mainly due to de novo production of new naïve T lymphocytes and reduced apoptosis [22, 24, 25]. Thus, the former process could explain enhanced immune reactivity to *M. tuberculosis* observed in the previous studies within the first year of HAART. In contrast, the functional immune response to *M. tuberculosis* in HIV/latent TB co-infected patients after prolonged HAART therapy has not been well studied. As a result, questions still remain regarding the extent and nature of the anti-mycobacterial immune reconstitution in the long-term of HAART. We therefore aimed at investigating the durability of HAART-driven anti-mycobacterial immune responses with the hypothesis that long-term HAART would still augment protective immune responses against *M. tuberculosis* in HIV/latent TB co-infected patients. In this study we observed an increased, but only partly, *M. tuberculosis*-specific functional immune responses in HIV/latent TB co-infected patients who received HAART for more than a year as compared to HAART-naïve patients.

## Methods

### Study participants

**Fig. 1:**
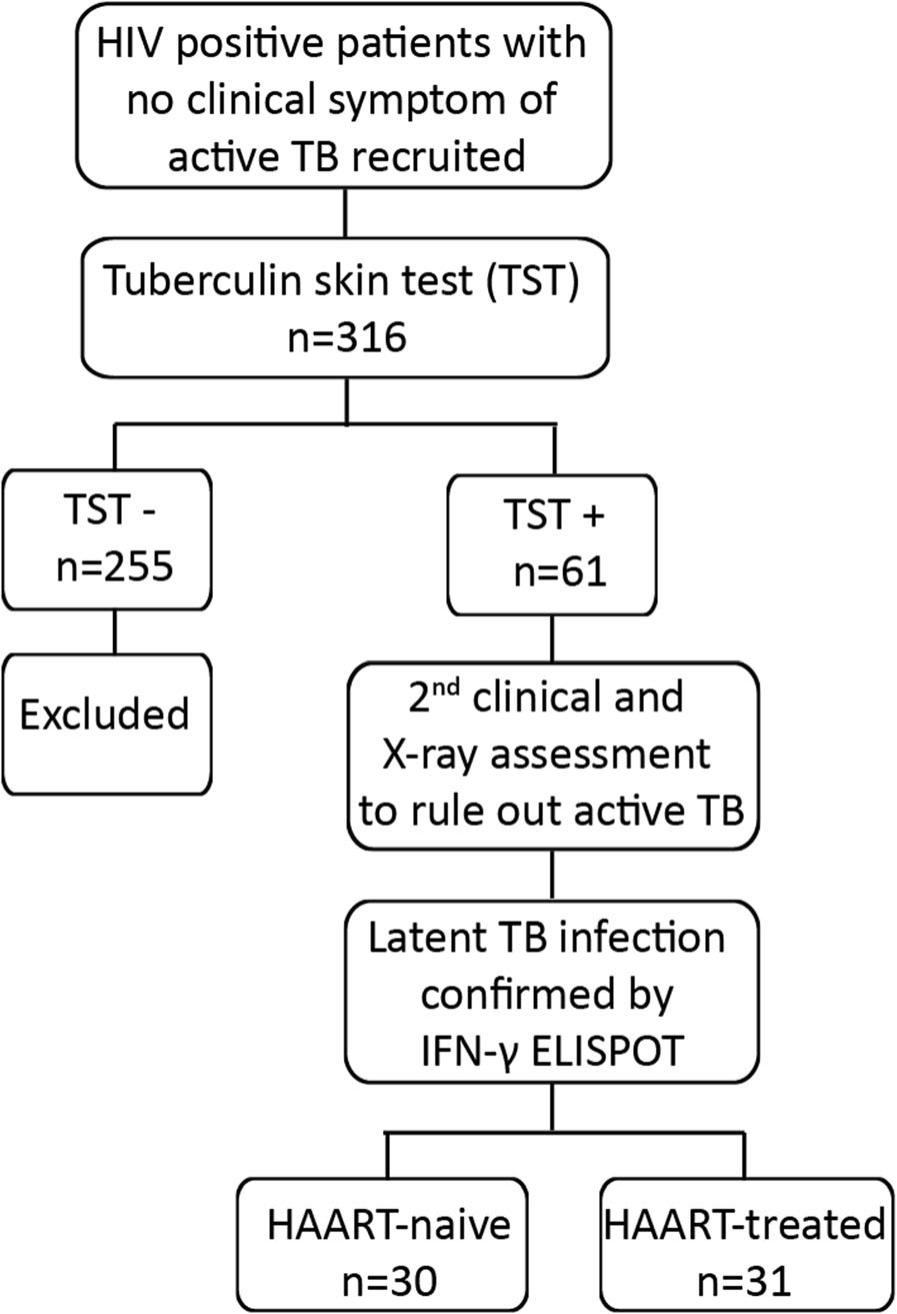
Flow chart of the recruitment and enrollment of participants in this study

The recruitment and enrollment of participants in this study is summarized in Fig. 1. HIV-infected patients were consecutively selected by their attending clinicians in ALERT hospital, Addis Ababa, Ethiopia from July 2011 to January 2012. Participants with active TB, active hepatitis, pregnancy, history of close contact with multidrug-resistant TB patients and prior Bacille Calmette-Guérin (BCG) vaccination within the previous 10 years were excluded from recruitment in this study. The exclusion of the relatively recent BCG vaccinated recipients was done to minimize impact of BCG on TST responses. Three hundred sixteen HIV positive patients were screened with the tuberculin skin test (TST) by administering 0.1 ml of the 2-TU PPD, RT23 (Statens Serum Institute (SSI), Denmark) intradermally. The transverse diameter of induration was measured after 48–72 h and the TST response was considered positive when the induration was > 5 mm [26, 27]. Of the 316 participants, only 61 were positive for TST and later confirmed by IFN-γ enzyme-linked immunospot (ELISPOT) to be latently infected with *M. tuberculosis*. Of these 61 participants, 30 were HAART-naïve and 31 received HAART for at least 1.5 up to 9 years, and were included in this cross-sectional study to evaluate *M. tuberculosis*-specific functional immune responses in HIV/latent TB co-infected patients with and without long-term HAART. As per then existing policy of the Ministry of Health of Ethiopia, all HAART-treated HIV positive individuals were presumably put on HAART when their CD4^+^ T cell counts were 200 cells/μl or less.

### Blood collection for CD4^+^ T cell count and PBMC isolation

Heparinized blood samples were collected from all study participants. CD4^+^ T cell count was determined by a Becton Dickinson (BD) FACSCount flow cytometer (BD Biosciences, USA) and PBMCs were also isolated by means of Ficoll-Paque PLUS™ (GE Healthcare Biosciences AB, Sweden) density centrifugation using Leucosep tubes (Greiner Bio-One B. V, Netherlands). PBMCs were stored until analysis at − 80 °C at a 5-10x10^6^cells/ml/vial in freezing media (10% Dimethyl sulfoxide (DMSO) and 90% Fetal Bovine Serum (FBS)) (Sigma-Aldrich Co, USA). When required, PBMCs were then thawed and re-suspended in RPMI 1640 supplemented with 1% Penicillin/Streptomycin, 1% L-Glutamine (Sigma-Aldrich Chemie GmbH, Germany) and 10% FBS (Sigma-Aldrich Co, USA).

### Ex vivo IFN-γ and IL-2 ELISPOT

Human IFN-γ and IL-2 ELISPOT kits (Mabtech AB, Sweden) were used to determine the frequency of IFN-γ and IL-2 secreting cells in response to *M. tuberculosis* antigens and were performed according to the manufacturer’s protocol and as described before [28]. Plates were seeded with 2 × 10^5^ PBMCs/well in duplicate in the presence of PPD, ESAT-6 (SSI, Denmark), anti-CD3 (positive control; Mabtech AB, Sweden) or left unstimulated (negative control). The final concentration of 5 μg/ml for PPD and ESAT-6, and 1:1000 dilution for anti-CD3 were used. The numbers of spot forming cells (SFCs) in respective wells were quantified using an automated ELISPOT plate reader (Autoimmun Diagnostika (AID), Germany). The intensity and size of the spots were predefined and the same setting was used throughout. The average SFC counts of the duplicate wells were calculated and the final number of antigen specific SFCs were determined by subtracting media background spots from those of stimulant containing wells. To reveal the validity of the test results, ELISPOT response was predefined to be at least 750 SFCs/10^6^PBMCs in the anti-CD3 positive control wells [29] and all results were valid. A positive IFN-γ response to *M. tuberculosis* antigen was taken as more than 50 SFCs/10^6^PBMCs after negative control well SFC subtraction [29, 30].

### T cell proliferation assay

Cell proliferation was determined by the carboxyfluorescein diacetate succinimidyl ester (CFSE) dilution assay using the CellTrace™ CFSE Cell Proliferation Kit (Invitrogen, USA) and was performed according to the manufacturer’s protocol. 2 × 10^6^ PBMCs were stained with final concentration of 0.3 μM CFSE prior to culture. Then, 1.5 × 10^5^ cells were cultured in a 96-wells plate in duplicate in the presence of PHA (positive control; Sigma-Aldrich Chemie GmbH, Germany), PPD and ESAT-6 (SSI) or left unstimulated (negative control) at 37 °C in a 5% CO_2_ humidified incubator. The final concentration of each antigens were 5 μg/ml. After 4 days (PHA) and 6 days (other conditions), the cells were harvested and stained with anti-CD3-APC, anti-CD4-PeCy5 and anti-CD8-PE (BD Biosciences). Cells were acquired by a FACSCanto II flow cytometer and data were analyzed by FlowJo (TreeStar, USA). Proliferating cells were defined as those with reduced (low or dim) CFSE expression and % specific proliferation defined as the fraction of cells with low CFSE after subtraction of unstimulated control cell values.

### Statistical analysis

Data were entered, cleaned and analyzed using SPSS version 15.0 and Graph pad Prism version 6.01 softwares. The normality of the data was evaluated by both Kolmogorov-Smirnov test and D’Agostino-Pearson normality test, and none of the continuous variables were Gaussian even after normalization. Thus, the frequencies of IFN-γ and IL-2 secreting cells responding to *M. tuberculosis* antigens between HAART-naïve and-treated groups were compared using a non-parametric Mann-Whitney test. Data are shown as median with interquartile range (IQR). Spearman correlation test was also used to demonstrate the association between two continuous variables. *P* < 0.05 were considered statistically significant.

## Results

### Demographic and clinical information

Sixty-one HIV-infected patients with latent TB infection were enrolled for the purpose this study and of these, 30 were HAART-naïve and 31 received HAART for more than a year. The majority, 49/61 (80.3%), of the enrolled participants were females and of these, 24 (49%) were HAART-naïve and 25 (51%) received HAART. The duration of therapy in the HAART-treated group ranged from 1.5 to 9 years with a median of 3 years (IQR 2.5–5). The median age of the participants was 32 years (IQR 29–40) with the highest proportion (24/61, 39%) in the age range of 30–39, of which 13/24 and 11/24 were HAART-naïve and HAART-treated respectively. Education wise, 39.3% (24/61) of the study participants had a primary level of education and 31.1% (19/61) were illiterate. The majority of participants, 60.7% (37/61) were married while 41.0% (25/61) were private workers and 36.1% (22/61) were housewives. In addition, hierarchal multiple regression analysis was performed to assess the association between demographic characteristics and antigen-specific IFN-γ and IL-2 responses. We observed that there were no statistical significant association between the demographic characteristics (age, gender, occupation, educational level, marital status) of the study participants, and the frequency of IFN-γ and IL-2 secreting cells irrespective of the stimuli antigen.

Thirteen participants (21.3%) had previously been vaccinated with BCG at childhood; of these, 2/13 (15.4%) were HAART-naïve and 11/13 (84.6%) HAART treated participants (Table 1). In addition, according to their hospital follow-up records, only 6 (5 HAART-naïve and 1 HAART-treated) had infections within 3 months of recruitment to this study. Upper respiratory tract Infections, urinary tract infections and sexually transmitted infections were the infections documented in the charts of these study participants.

**Table 1:** Clinical and laboratory data of the study participants (*n* = 61)

Variable	No. (%) HIV/latent TB co-infected participants		
	HAART-naïve *n* = 30	HAART-treated *n* = 31	Total *n* = 61
CD4^+^ count (cells/μl), Median (IQR)	445 (371–626)	502 (363–564)	477 (367–567)
201–350	7 (23.3)	4 (12.9)	11 (18.0)
351–500	10 (33.3)	11 (35.5)	21 (34.4)
>500	13 (43.3)	16 (51.6)	29 (47.5)
^a^History of Previous BCG Vaccination			
Yes	2 (6.7)	11 (35.5)	13 (21.3)
No	28 (93.3)	20 (64.5)	48 (78.7)
^b^Recent infections			
Present	5 (16.7)	1 (3.2)	6 (9.8)
Absent	25 (83.3)	30 (96.8)	55 (90.2)

^a^Prior BCG vaccination more than 10 years before the study; ^b^ within 3 months before recruitment into study

### CD4^+^ T cell count

We determined the CD4^+^ T cell count of enrolled participants as shown in Table 1. All study participants had CD4^+^ T cell counts greater than 200/μl with the overall median count of 477/μl (IQR 367–567). CD4^+^ T-cell count of HAART-treated participants was relatively higher than HAART-naïve group with a median count of 502/μl (IQR 363–564) and 445/μl (IQR 371–626) respectively, though these differences did not reach statistical significance, *p* = 0.9368. Nevertheless, we observed that HAART-treated participants considerably gained CD4^+^ T cells years after therapy (Additional file 3: Figure S3a). Based on the CD4^+^ T cell count, study participants were divided into 3 subgroups: 201–350/μl, 351–500/μl and > 500/μl. Of the total participants, 29 (47.5%) and 21 (34.4%) had a CD4^+^ T-cells of > 500/μl and 351–500/μl respectively. 43.3% (13/30) and 51.6% (16/31) of HAART-naïve and HAART-treated participants respectively had a CD4^+^ T-cell count of > 500 cells/μl.

### Frequency of IFN-γ secreting cells in response to *M. tuberculosis* antigens

**Fig. 2:**
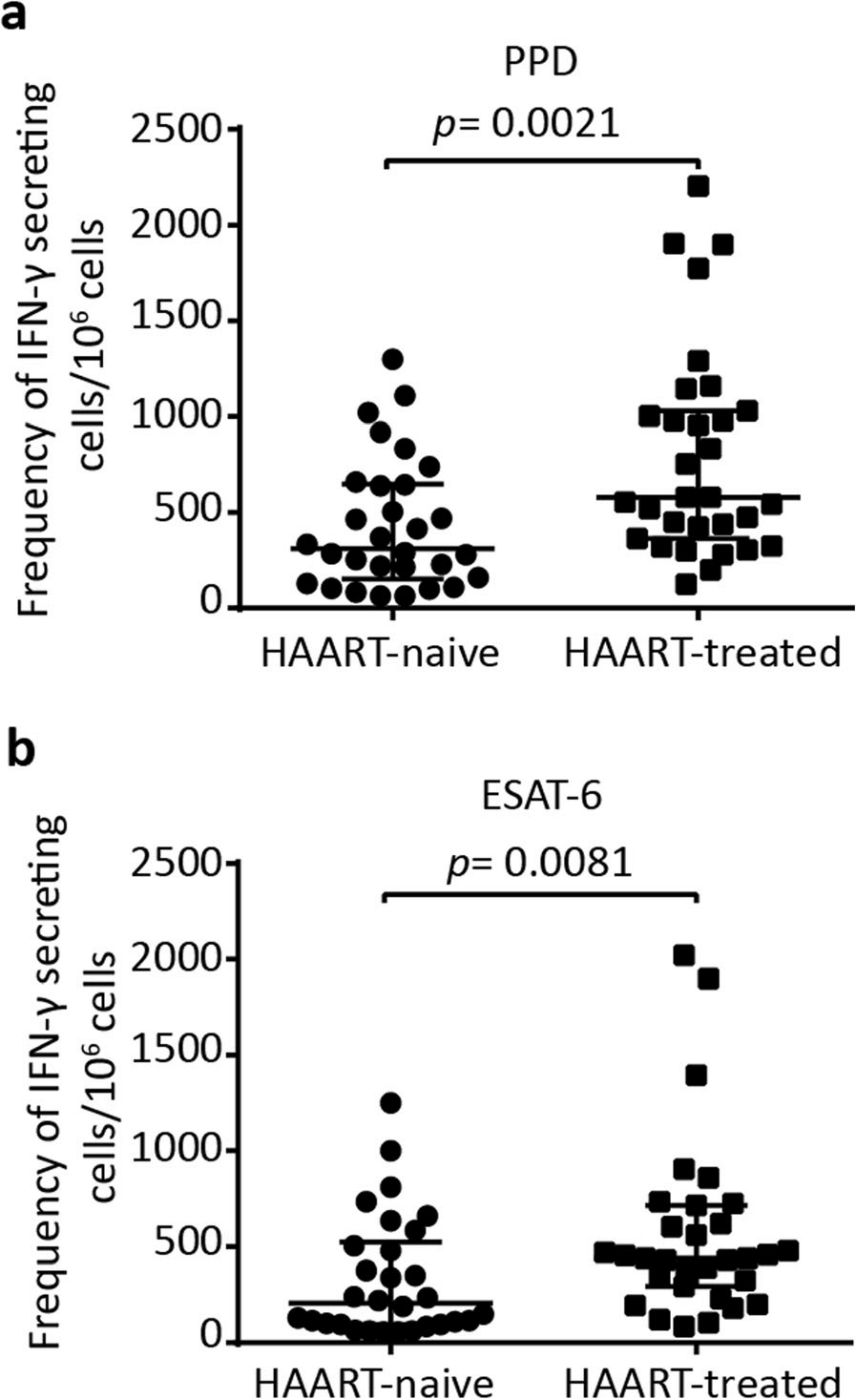
Frequency of IFN-γ secreting cells in response to *M. tuberculosis* antigens. The frequency of IFN-γ secreting cells responding to PPD (**a**) and ESAT-6 (**b**) in HAART-naïve (*n* = 30) and HAART-treated (*n* = 31) participants. *p* value was determined using the Mann-Whitney test. Median with interquartile range (IQR) is shown

PPD and ESAT-6 specific IFN-γ responses were assessed in participants who received HAART for at least 1.5 years in comparison with those who were HAART-naïve. As shown in Fig. 2a, the median frequency of PPD-specific IFN-γ producing cells was significantly higher in HAART-treated participants (580 SFCs/10^6^PBMCs, IQR 365–2205) as compared to the pre-HAART participants (313 SFCs/10^6^PBMCs, IQR 153–1300), *p* = 0.0021. In addition, the median frequency of IFN-γ secreting cells in response to ESAT-6 was also significantly higher in the HAART-treated group (440 SFCs/10^6^PBMCs, IQR 295–2020) than HAART-naive subjects (205 SFCs/10^6^PBMCs, IQR 95–1250), *p* = 0.0081 (Fig. 2b). We then used spearman correlation analysis to determine whether *M. tuberculosis*-specific IFN-γ responses are associated with CD4^+^ T cell count of the patients. Regardless of HAART status, CD4^+^ T cell counts were not significantly correlated with the frequency IFN-γ secreting cells responding to PPD and ESAT-6 (Additional file 1: Figure S1). This data demonstrates an increased *M. tuberculosis*-specific IFN-γ response in HIV/latent TB co-infected patients with long-term HAART irrespective of the CD4^+^ T cell count.

### Frequency of IL-2 secreting cells in response to *M. tuberculosis* antigens

**Fig. 3:**
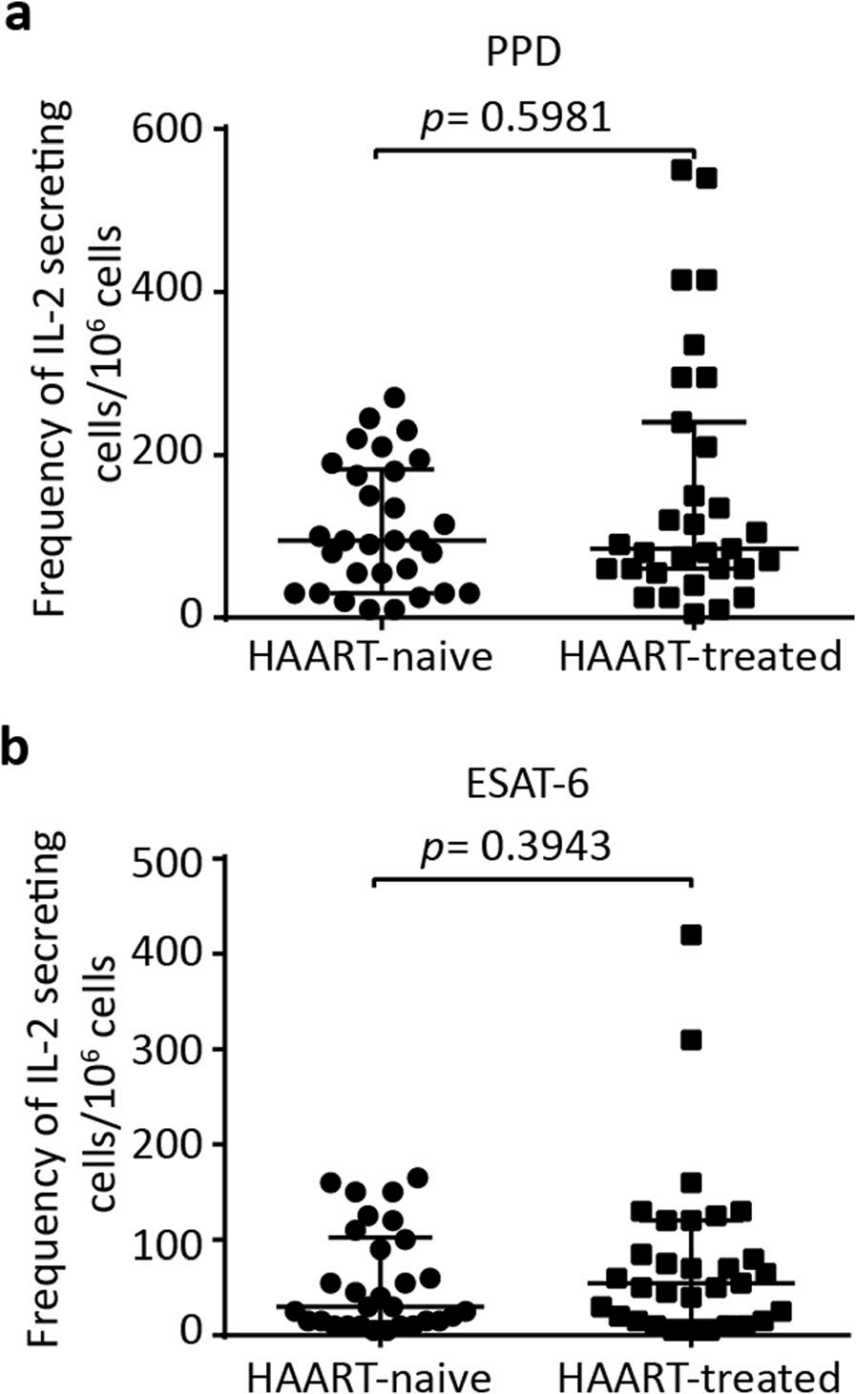
Frequency of IL-2 secreting cells in response to *M. tuberculosis* antigens. The frequency of IL-2 secreting cells responding to PPD (**a**) and ESAT-6 (**b**) in HAART-naïve (*n* = 30) and HAART-treated (*n* = 31) participants. *p* value was determined using Mann-Whitney test. Median with interquartile range (IQR) is shown

We then quantified the frequencies of IL-2-secreting cells responding to PPD and ESAT-6 to determine the effect of anti-retroviral treatment on the *M. tuberculosis* antigen-specific IL-2 response. There was no significant difference in the IL-2 response to PPD between HAART-naïve (median 95 SFCs/10^6^PBMCs, IQR 30–183) and HAART-treated (median 85 SFCs/10^6^PBMCs, IQR 60–240) groups, *p* = 0.5981 (Fig. 3a). Similarly, ESAT-6 elicited IL-2 responses did not differ significantly between HAART-naïve (median 30 SFCs/10^6^PBMCs, IQR 14–103) and HAART-treated (median 55 SFCs/10^6^PBMCs, IQR 15–120) subjects, *p* = 0.3943 (Fig. 3b). Similar to the IFN-γ responses, there was no statistically significant correlation between CD4^+^ T cell count and the frequencies of IL-2 secreting cells responding to PPD or ESAT-6 irrespective of anti-retroviral treatment (Additional file 2: Figure S2). This shows that *M. tuberculosis*-specific IL-2 response was not enhanced during the long-term HAART therapy regardless of the CD4^+^ T cell count.

### Correlation of IFN-γ and IL-2 responses specific to *M. tuberculosis* antigens

**Fig. 4:**
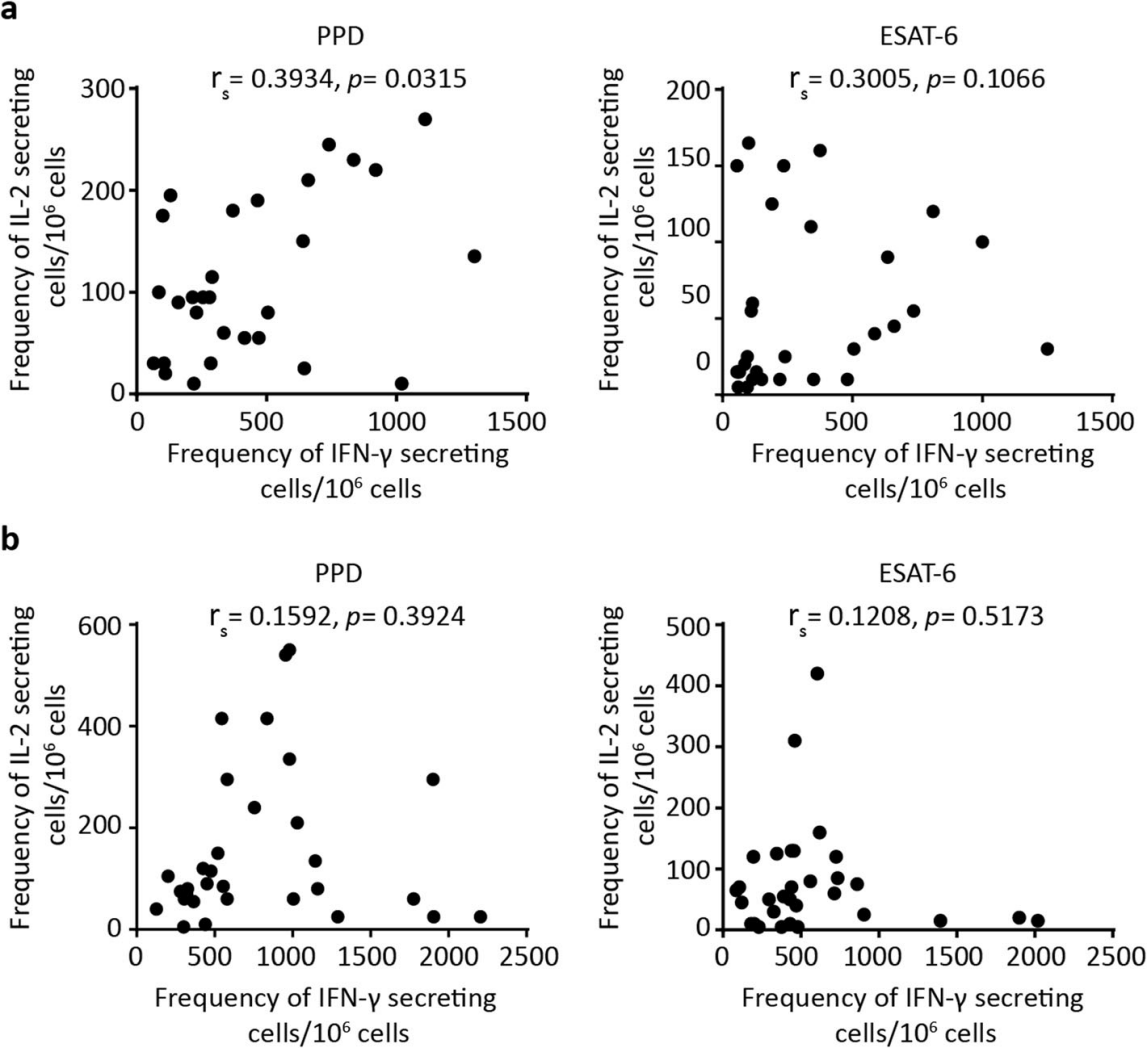
Correlation of IFN-γ and IL-2 responses specific to *M. tuberculosis* antigens. Correlation between the frequency PPD and ESAT-6 specific IFN-γ and IL-2 secreting cells in HAART-naïve (**a**, *n* = 30) and HAART-treated (**b**, *n* = 31) participants. Spearman correlation was used to calculate correlation coefficients (r_s_) and *p* values

Correlation between the *M. tuberculosis* antigen-specific frequencies of IFN-γ and IL-2-secreting cells was assessed. In pre-HAART patients (Fig. 4a), we found a statistically significant correlation between PPD-specific frequencies of IFN-γ and IL-2 secreting cells (r_s_ = 0.3934, *p* = 0.0315) and a modest though not statistically significant correlation in the ESAT-6 stimulated cells (r_s_ = 0.3005, *p* = 0.1066). In contrast, the IFN-γ and IL-2 responses were unrelated when elicited to either PPD (r_s_ = 0.1592, *p* = 0.3924) or ESAT-6 (r_s_ = 0.1208, *p* = 0.5173) in the HAART-treated group (Fig. 4b). Thus, the absence of correlation between *M. tuberculosis*-specific IFN-γ and IL-2 productions in the HAART-treated participants suggest that these cytokines could be secreted by different T cell subsets in patients on therapy.

### Correlation of *M. tuberculosis* antigen-specific cytokine responses and duration of HAART

**Fig. 5:**
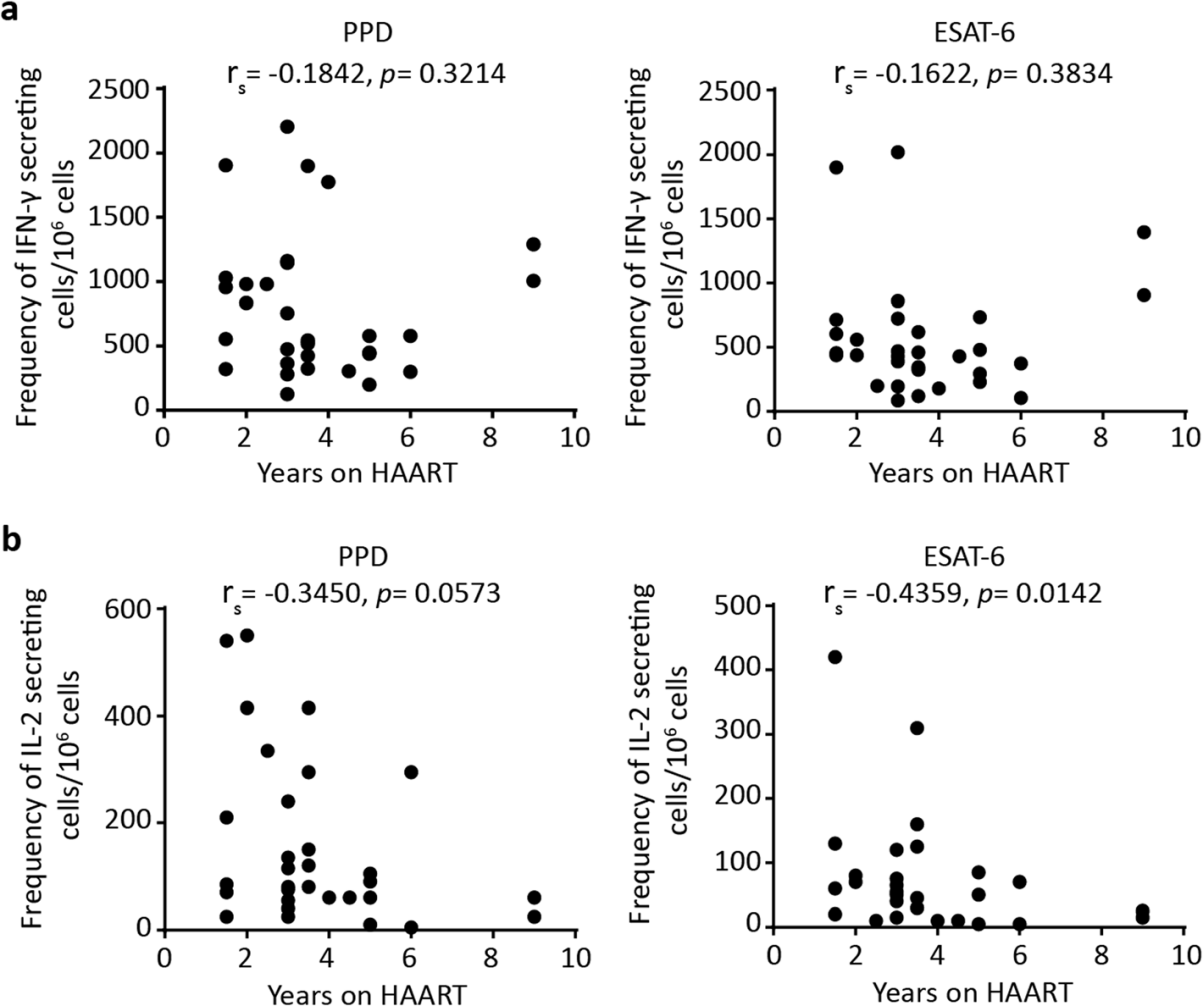
Correlation of *M. tuberculosis* antigen-specific cytokine responses and duration of HAART. The correlation between PPD and ESAT-6 specific frequency of IFN-γ (**a**) and IL-2 (**b**) secreting cells and duration of HAART among the HAART-treated participants (*n* = 31). Spearman correlation was used to calculate correlation coefficients (r_s_) and *p* values

We then assessed whether IFN-γ and IL-2 responses specific to *M. tuberculosis* antigens were associated with duration of HAART. No association was observed between duration of HAART and IFN-γ production responding to PPD (r_s_ = − 0.1842, *p* = 0.3214) or ESAT-6 (r_s_ = − 0.1622, *p* = 0.3834) (Fig. 5a), nor were there correlations between therapy duration and IFN-γ/IL-2 ratios to PPD (r_s_ = 0.2466, *p* = 0.1812) or ESAT-6 (r_s_ = 0.2568, *p* = 0.1632) (data not shown). Importantly and in stark contrast, we observed that *M. tuberculosis-*specific IL-2 responses negatively correlated with duration on antiretroviral therapy; this relation was apparent for both ESAT-6 (r_s_ = − 0.4359, *p* = 0.0142) and PPD (r_s_ = − 0.3450, *p* = 0.0573) (Fig. 5b).

We considered the possibility that this finding might be explained by a drop in IL-2 responses related to a loss in CD4^+^ T cells in some patients on prolonged HAART after initial gains. Such a possibility would predict a positive correlation between net CD4^+^ T cell gain after therapy and IL-2 production. The number of CD4^+^ T cells gained after HAART was positively associated with therapy duration (Additional file 3: Figure S3b). However, as depicted in the Additional file 3: Figure S3c and d, neither IL-2 nor IFN-γ secreting cell frequencies were related to CD4^+^ T cell gain. Collectively, these results indicate that it is HAART duration but not CD4^+^ T cell count nor CD4^+^ T cell gain which predicted decreased *M. tuberculosis*-specific IL-2 responses. In contrast, no such correlation was observed with HAART duration and IFN-γ responses.

### Association of IL-2 production and T cell proliferation in response to *M. tuberculosis* antigens

**Fig. 6:**
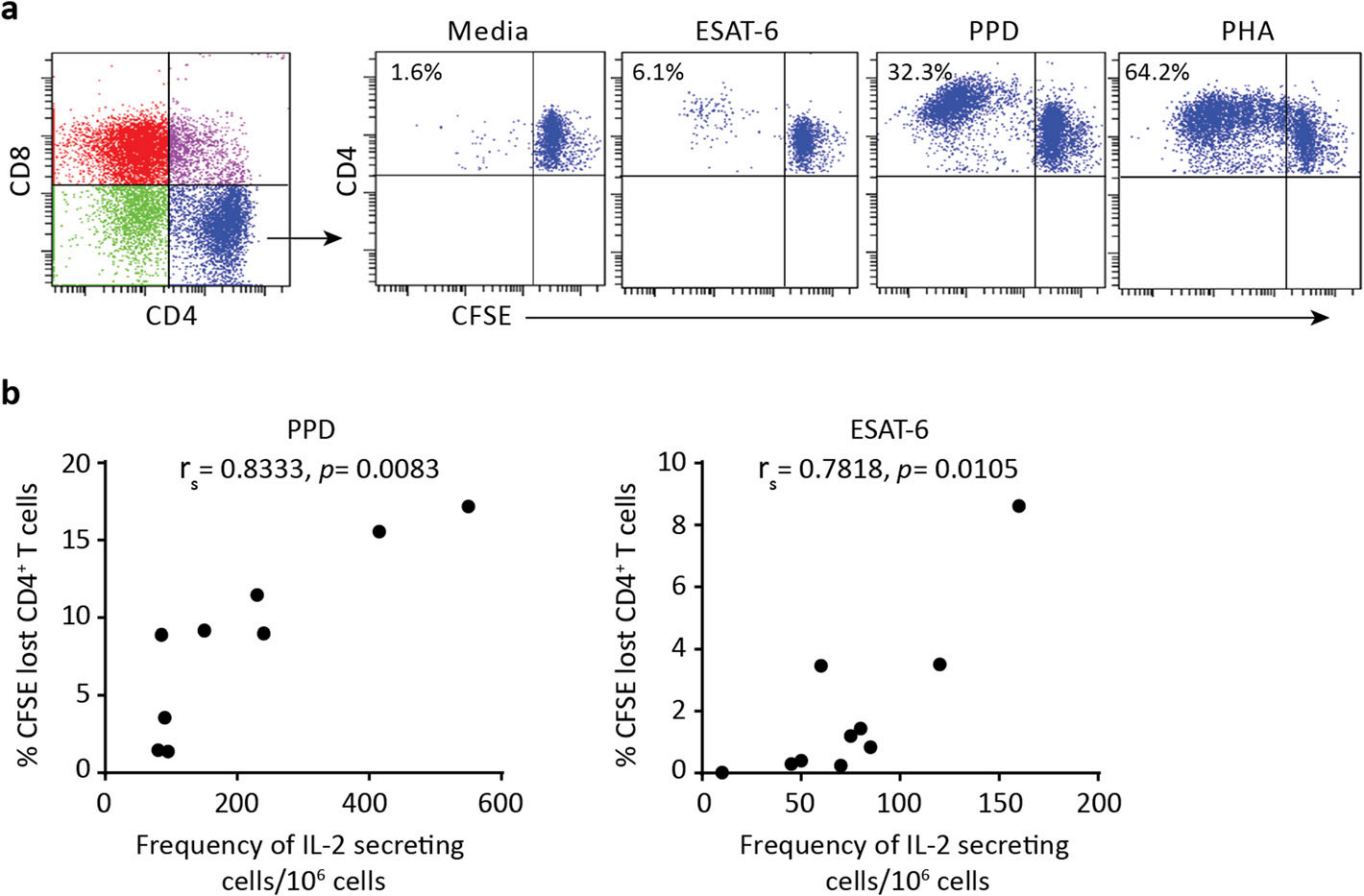
Association of IL-2 production and CD4^+^ T cell proliferation in response to *M. tuberculosis* antigens. **a** The gating strategy for CFSE lost proliferating CD4^+^ T cells in the Media (unstimulated control), ESAT-6, PPD and PHA (positive control) stimulated cells. Numbers indicate the percentage CFST lost cells among the CD4^+^ T cells. **b** Correlation between frequency of IL-2-secreting cells and proliferating CD4^+^ T cells among total PBMCs after stimulation with PPD (*n* = 9) and ESAT-6 (*n* = 10). Spearman correlation was used to calculate correlation coefficients (r_s_) and *p* values

IL-2 is a growth factor that promotes proliferation of the antigen specific T cells [11, 31]. To assess whether IL-2 production was associated with T cell proliferative capacity, we performed in vitro proliferation assays on a limited number of participants selected regardless of their HAART therapy. The proliferative capacity of the T cells and CD4^+^ T cells among the total PBMCs were assessed by flow cytometry using the gating strategy shown in Fig. 6a. We found no statistically significant association between the frequencies of IL-2 secreting cells and proliferating T cells in response to PPD (r_s_ = 0.3333, *p* = 0.3853) and ESAT-6 (r_s_ = 0.5758 *p* = 0.0883) (Additional file 4: Figure S4). However, after gating on CD4^+^ T cells, CFSE dim proliferating CD4^+^ T cells were positively correlated with the frequencies of IL-2 secreting cells with PPD (r_s_ = 0.8333, *p* = 0.0083) and ESAT-6 (r_s_ = 0.7818, *p* = 0.0105) stimulation (Fig. 6b). This confirms that *M. tuberculosis-*specific IL-2 response is highly correlated with the mycobacteria-specific CD4^+^ T cell proliferation in HIV/latent TB co-infected patients.

## Discussion

The incidence rate of TB in HIV-infected patients who initiate HAART is significantly decreased compared to patients without therapy, but even on antiretroviral therapy, the risk for TB is higher than HIV uninfected individuals [14, 32]. Clarification of the quality and quantity of the incomplete immune recovery and developing means to improve clinical outcomes thus remain an important priority in the HIV field. HAART-driven immune restoration is thought to occur in two phases; the first and fast phase is due to immediate redistribution of memory CD4^+^ T cells from lymphoid tissues to peripheral blood, but does not involve significant net changes in CD4^+^ T cell number, followed by slow gradual immune recovery as a result of de novo production of naïve or memory T lymphocytes and reduced apoptosis [21–25, 33, 34]. Most studies that have assessed restoration of *M. tuberculosis*-specific responses have typically involved cohort studies evaluating immune reconstitution before and after up to one year of therapy, which would be predicted to address changes primarily due to the fast redistribution phase. We carried out this cross-sectional study to enumerate TB-specific immune responses among patients without HAART and with a range of duration on therapy from 1.5 up to 9 years. We found that PPD and ESAT-6-specific IFN-γ responses were significantly higher in HIV-infected patients who received HAART for more than a year compared to the pre-HAART participants, consistent with the observations of cohort studies evaluating responses within the first 6 to 12 months of HAART [18, 35, 36]. Hence, this shows that anti-mycobacterial IFN-γ responses are restored after initiation of HAART and maintained for prolonged period of the therapy. In contrast to the IFN-γ response, however there was no significant difference between HAART-naive and-treated patients in *M. tuberculosis*-specific IL-2 production. These results contrast with studies done on patient cohorts with less than one year of HAART [37, 38]. Notably, we observed that *M. tuberculosis*-specific IL-2 responses were inversely correlated with duration of HAART, and not related to either CD4^+^ T cell count or CD4^+^ T cell gain after HAART. These findings raise the possibility that IL-2 responses may be restored only transiently during the initial redistribution phase, which would accommodate previous studies, but are unable to be maintained during long-term antiretroviral therapy in the secondary phase.

T cell subpopulations including CD4^+^ and CD8^+^ T cells are known to produce IFN-γ and/or IL-2. These T cells can be divided into distinct populations of effector cells, effector memory and central memory cells [39]. Effector T cells show immediate effector function of secreting IFN-γ whereas effector memory T cells produces both IFN-γ and IL-2 cytokines [40, 41], but in advanced HIV disease tend to produce relatively more IFN-γ [42, 43]. On the other hand, central memory T cells typically produce predominantly IL-2 [40, 41]. Presumably the differences we observed in IL-2 responses between the relatively early HAART and prolonged HAART reflect differences in the frequencies of these T cells subsets. However, since our studies were done on unfractionated cells, we could not enumerate the relative contribution of these subsets, nor the frequency of IFN-γ/IL-2 co-producing cells which may have impacted the observed results. Nonetheless, the fact that we did not observe correlations between the cytokine producing cells and CD4^+^ T cells counts, between IFN-γ and IL-2 responses in patients are findings consistent with the possibility that the IFN-γ and IL-2 responses measured here are being produced by different subsets of T cells. Thus, we would speculate that some subsets are preferentially induced or maintained at the expense of others in patients undergoing immune restoration after HAART. An example of how this might occur can be ascertained from known differential requirements of CD8^+^ and CD4^+^ T cell subsets for IL-7 and IL-15. In general, memory CD8^+^ T cells appear more dependent on IL-15 than IL-7, whereas memory CD4^+^ T cells may be more dependent on IL-7 [40, 41]. Consistent with these findings, CD4^+^ T cell counts are improved with IL-7 supplementation in humans on HAART [44] and in simian immunodeficiency virus (SIV)-infected and treated macaques, but not with IL-15 therapy in macaques [45, 46]. The findings of reduced IL-7 producing capacity of lymph node stromal cells associated with fibrosis in the para-cortical T cell zone [47], as well as improvements with anti-fibrotic therapy [48] contribute to the view that IL-7 may be particularly important in the maintenance of adequate memory CD4^+^ T cells or subsets thereof involved in IL-2 production. Deficiency of such cells could lead to suboptimal IL-2 production as we have observed here. Alternatively, failure to attain or maintain adequate IL-2 production may also reflect continued high rates of apoptosis of CD4^+^ T cells or their subsets [24, 25, 49]. Hence, further studies are required to investigate whether IL-7 and other factors are involved in restoration of IL-2 responses of *M. tuberculosis*-specific memory T cells in HIV infected patients receiving HAART.

Regardless of mechanisms of immune memory restoration or homeostatic maintenance, it is likely that *M. tuberculosis*-specific T cell production of both IFN-γ and IL-2 is important for optimal prevention of reactivation of latent TB infection. The necessity for IFN-γ is aptly demonstrated by the increased risk of TB and other mycobacterial diseases in individuals with genetic defects in the production or action of IFN-γ [50], yet IFN-γ is clearly not sufficient because most TB patients readily produce IFN-γ. IL-2 promotes the expansion of the antigen specific T cells and likely play an indispensable role in control of the *M. tuberculosis* infection [11, 31, 51]. Consistent with the role of IL-2 in proliferation, we confirmed on a subset of the patients in this study that *M. tuberculosis*-specific IL-2 response correlated positively with CD4^+^ T cell proliferation ex vivo*.* The negative correlation of IL-2 production with duration of HAART suggests that proliferative capacity of *M. tuberculosis*-specific CD4^+^ T cells in HIV-infected patients could be diminished over time after antiretroviral therapy regardless of the gain in CD4^+^ T cell count. Hence, a failure to maintain adequate levels of *M. tuberculosis*-specific IL-2 secreting cells in patients on long-term HAART, predicts that such patients, despite circulating levels of IFN-γ producing cells, may be impaired in the development or augmentation of adaptive immune responses to either reactivated latent TB or new TB infections.

Despite the evidence of the importance of IL-2 for adequate immune responses, a recent prospective study in South Africa evaluating the ability of cytokine levels to predict risk of TB observed that TB-specific IL-2 detected in vitro was actually higher among patients who eventually developed disease [52]. This observation is not consistent with what we would have predicted although we did not compare cytokine production in the latent TB infection with active TB in the present study. These findings serve as a reminder that, like IFN-γ, levels of IL-2 and other cytokines, even if presumed necessary for protection, do not necessarily predict disease. It further underscores the importance of prospective studies to correlate immune responses with actual TB disease.

The cross-sectional approach we have utilized here has the advantage that patients on long-term HAART can be more conveniently recruited and evaluated, but the limitation is that, unlike prospective studies, no baseline values prior to HAART were tested. Thus, we cannot rule out the possibilities that prior to HAART, those patients on long-term HAART had lower levels of IL-2 than those of early HAART subjects, and that either IL-2 increased proportionally with IFN-γ in the HAART period similarly in both groups, or that IL-2 never changed in the long-term HAART group. In addition to baseline testing, an IL-2 assay of greater sensitivity than the currently available are required since the levels of most participants almost approached the limit of detection. In any case, it is clear that definitive testing of the hypothesis that IL-2 is only transiently increased during the fast redistribution phase of immune reconstitution, but not sustained during the long-term HAART, requires both a prospective study evaluating patients at baseline, and a much longer follow-up period than has previously been employed, and one which, like the aforementioned South African study evaluates disease outcomes.

## Conclusion

In summary, this study has shown an increased anti-mycobacterial immune response in HIV/latent TB co-infected patients with long-term HAART as evaluated by IFN-γ response. However, the failure of *M. tuberculosis*-specific IL-2 responses to improve may indicate that patients on therapy remain only partly competent to mount and augment an effective *M. tuberculosis*-specific response. This finding is in keeping with the observation that patients on long-term HAART remain at risk, albeit reduced, for new opportunistic infections, and require careful monitoring and follow-up. These findings also call for more prospective studies which evaluate subsets of T cells in both pre-HAART and over a much longer time period of the therapy to more accurately reveal the immune potential and ability to protect against disease of patients both during the short-term and long-term phases of immune reconstitution.

## Supplementary information

**Additional file 1: Figure S1.** Correlation of *M. tuberculosis* antigen-specific IFN-γ responses and CD4^+^ T cell count. Correlation between frequency IFN-γ secreting cells responding to PPD or ESAT-6, and the CD4^+^ T cell count in HAART-naïve (**a**, *n* = 30) and HAART-treated (**b**, *n* = 31) participants. Spearman correlation was used to calculate correlation coefficients (r_s_) and *p* values.

**Additional file 2: Figure S2.** Correlation of *M. tuberculosis* antigen-specific IL-2 responses and CD4^+^ T cell count. Correlation between frequency IL-2 secreting cells responding to PPD or ESAT-6, and the CD4^+^ T cell count in HAART-naïve (**a**, *n* = 30) and HAART-treated (**b**, *n* = 31) participants. Spearman correlation was used to calculate correlation coefficients (r_s_) and *p* values.

**Additional file 3: Figure S3.** CD4^+^ T cell count increased with therapy duration after HAART but not correlated with *M. tuberculosis* specific IL-2 nor IFN-γ responses. (**a**) CD4^+^ T cell count before and after HAART in HAART-treated participants (*n* = 23). *p* value was determined using Mann-Whitney test. (**b**) Correlation between the number of increased CD4^+^ T cells with HAART and duration of therapy. Correlation of the number of CD4^+^ T cells gained after therapy with PPD and ESAT-6 specific IL-2 (**c**) and IFN-γ (**d**) responses in HAART-treated participants (*n* = 23). Spearman correlation was used to calculate correlation coefficients (r_s_) and *p* values.

**Additional file 4: Figure S4.** Association of IL-2 production and T cell proliferation in response to *M. tuberculosis* antigens. Correlation between frequency of IL-2-secreting cells and proliferating T cells among total PBMCs after stimulation with PPD (*n* = 9) and ESAT-6 (*n* = 10). Spearman correlation was used to calculate correlation coefficients (r_s_) and *p* values.

## Data Availability

The datasets used and/or analyzed during the current study are available from the corresponding author on reasonable request.

## Abbreviations

AHRI: Armauer Hansen Research Institute
AID: Autoimmun Diagnostika
BCG: Bacille Calmette-Guérin
CD: Cluster of differentiation
CSFE: Carboxyfluorescein diacetate succinimidyl ester
DMSO: Dimethyl sulfoxide
ELISPOT: Enzyme-linked immunospot
ESAT-6: Early secreted antigen-6
FBS: Fetal Bovine Serum
HAART: Highly active anti-retroviral therapy
HIV: Human immunodeficiency virus
IFN: Interferon
IL: interleukin
IQR: Interquartile range
*M. tuberculosis*: *Mycobacterium tuberculosis*
PBMC: Peripheral blood mononuclear cell
PHA: Phytohemagglutinin
PPD: Purified protein derivative
SFC: Spot forming cell
SIV: Simian immunodeficiency virus
SSI: Statens Serum Institute
TB: Tuberculosis
TNF: Tumor necrosis factor
TST: Tuberculin skin test
