# Supplementary figures and images for: Enhanced IFN-γ, but not IL-2, response to Mycobacterium tuberculosis antigens in HIV/latent TB co-infected patients on long-term HAART
Source: BMC Immunol. 2019 Oct 11;20:35. doi: 10.1186/s12865-019-0317-9 (PMC6788090; doi:10.1186/s12865-019-0317-9)

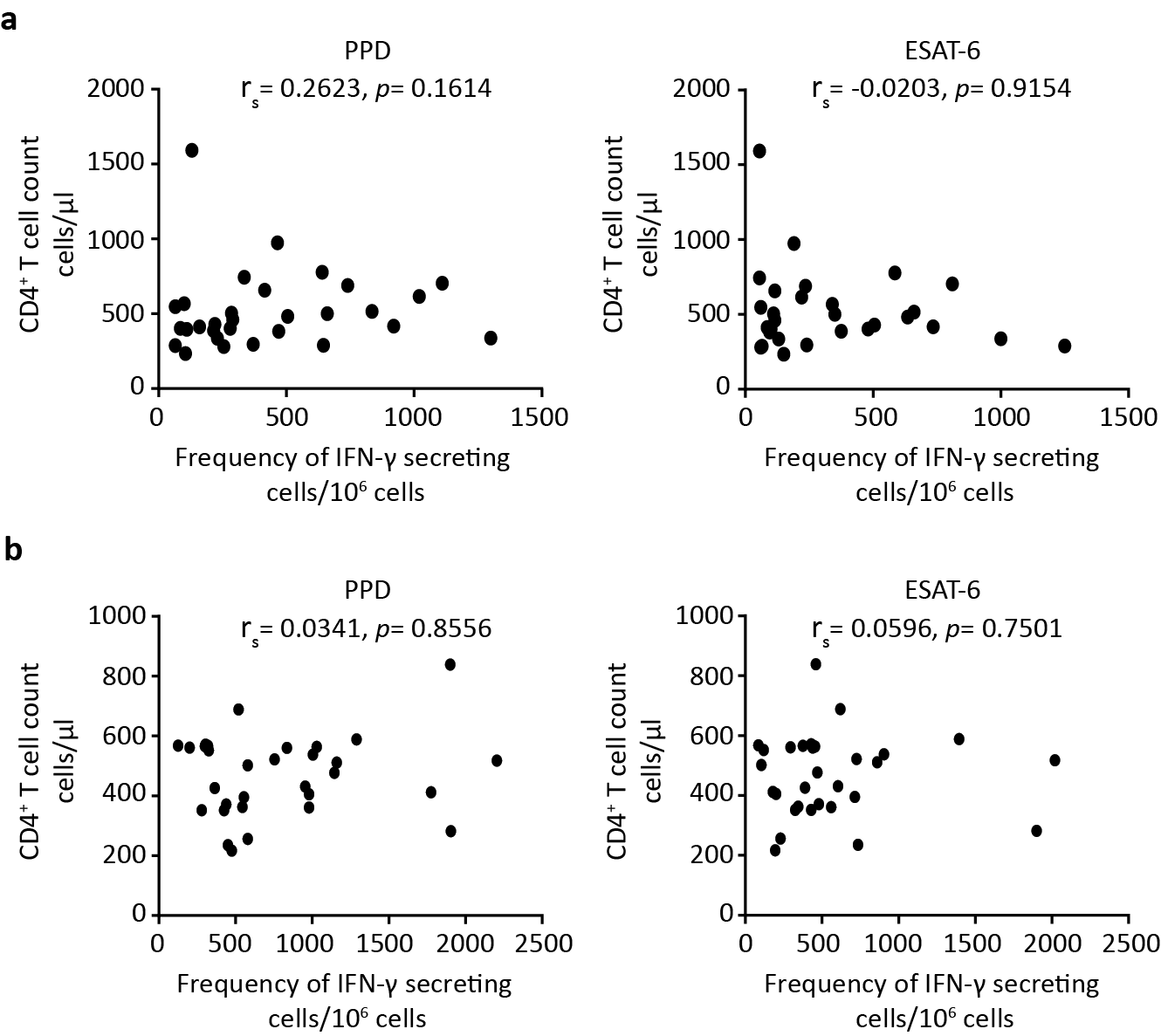

Supplement: Supplementary file 1 — Additional file 1: Figure S1. Correlation of M. tuberculosis antigen-specific IFN-γ responses and CD4+ T cell count. Correlation between frequency IFN-γ secreting cells responding to PPD or ESAT-6, and the CD4+ T cell count in HAART-naïve (a, n = 30) and HAART-treated (b, n = 31) participants. Spearman correlation was used to calculate correlation coefficients (rs) and p values. [file 12865_2019_317_MOESM1_ESM.png]

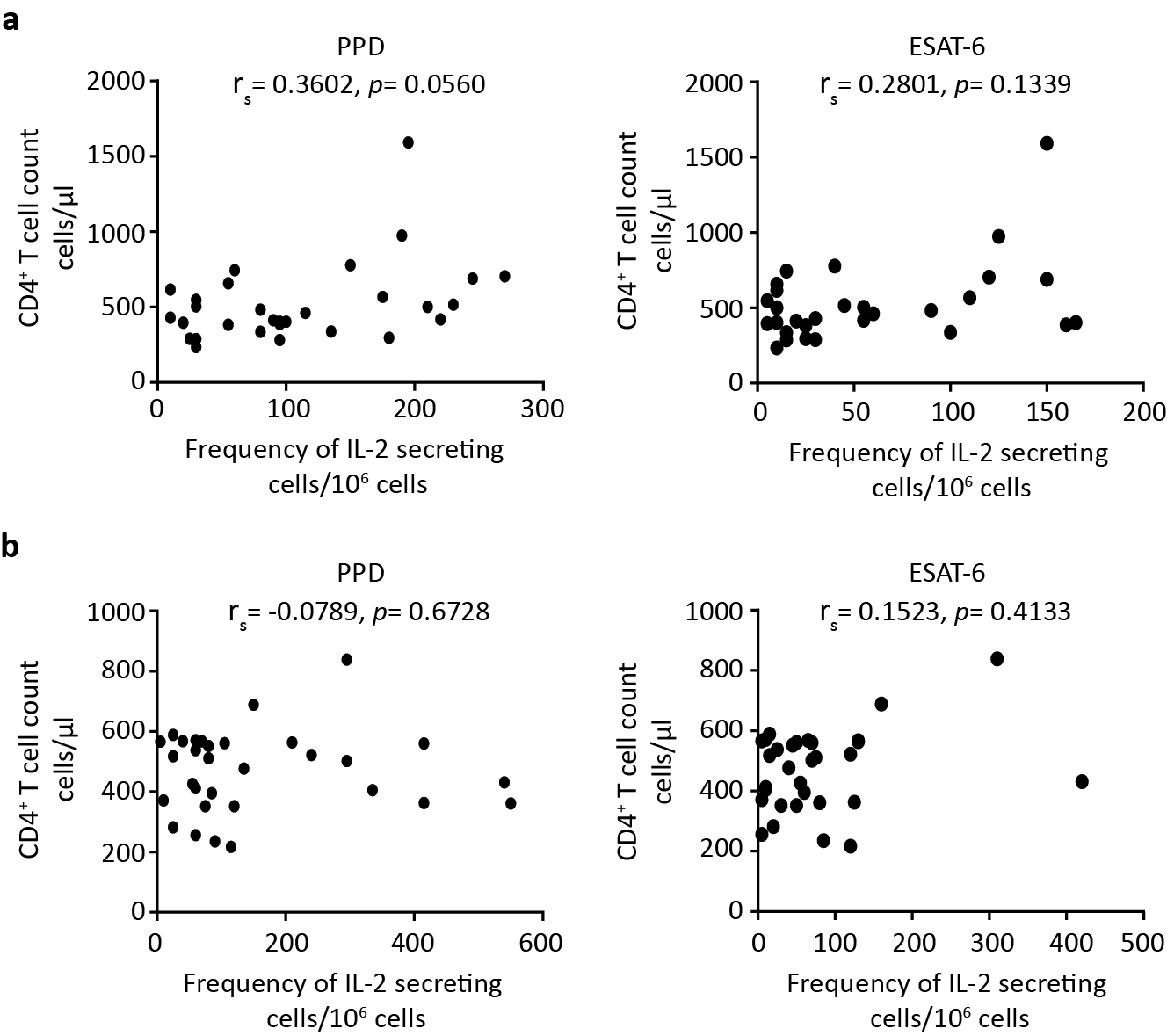

Supplement: Supplementary file 2 — Additional file 2: Figure S2. Correlation of M. tuberculosis antigen-specific IL-2 responses and CD4+ T cell count. Correlation between frequency IL-2 secreting cells responding to PPD or ESAT-6, and the CD4+ T cell count in HAART-naïve (a, n = 30) and HAART-treated (b, n = 31) participants. Spearman correlation was used to calculate correlation coefficients (rs) and p values. [file 12865_2019_317_MOESM2_ESM.png]

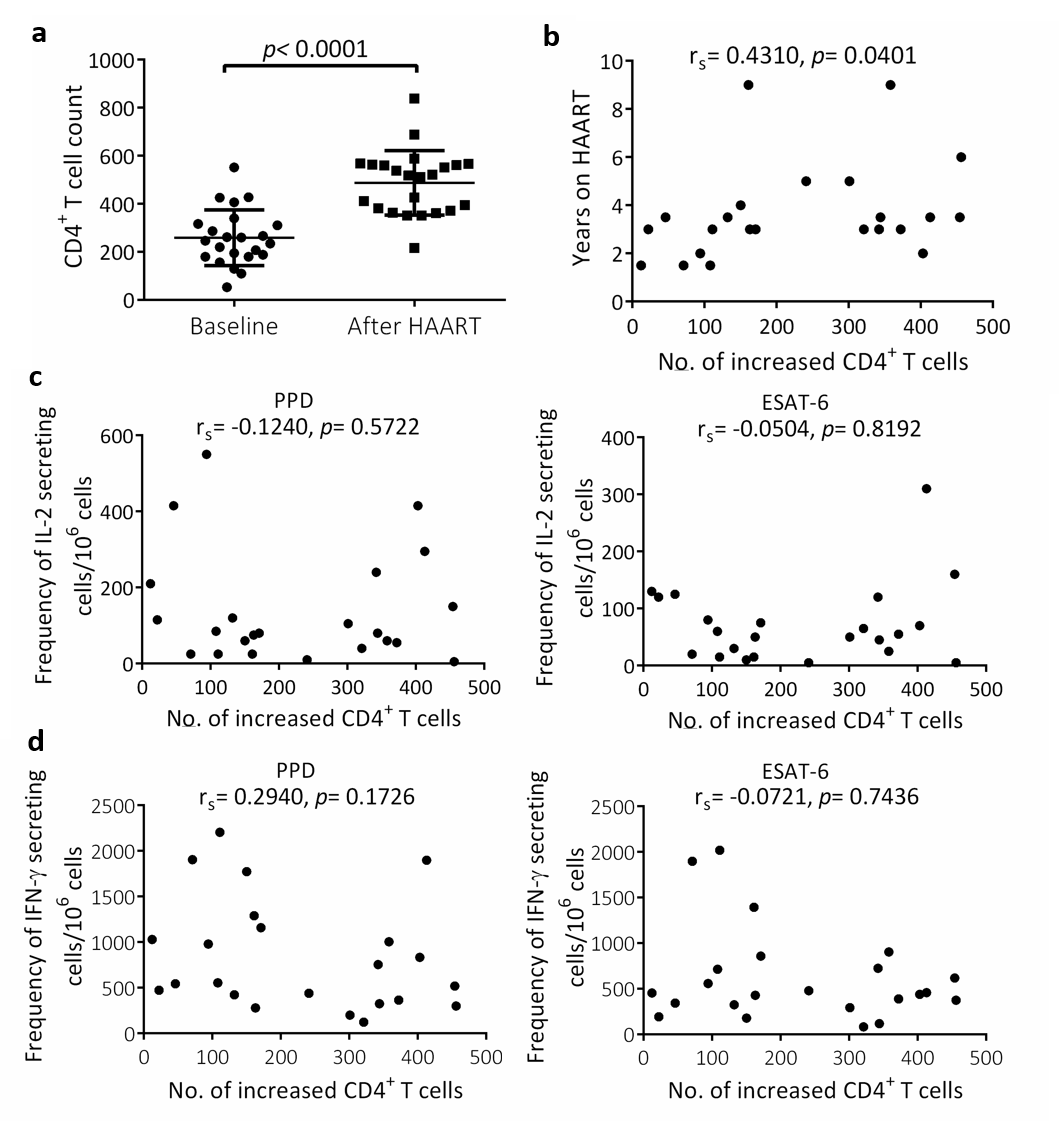

Supplement: Supplementary file 3 — Additional file 3: Figure S3. CD4+ T cell count increased with therapy duration after HAART but not correlated with M. tuberculosis specific IL-2 nor IFN-γ responses. (a) CD4+ T cell count before and after HAART in HAART-treated participants (n = 23). p value was determined using Mann-Whitney test. (b) Correlation between the number of increased CD4+ T cells with HAART and duration of therapy. Correlation of the number of CD4+ T cells gained after therapy with PPD and ESAT-6 specific IL-2 (c) and IFN-γ (d) responses in HAART-treated participants (n = 23). Spearman correlation was used to calculate correlation coefficients (rs) and p values. [file 12865_2019_317_MOESM3_ESM.png]

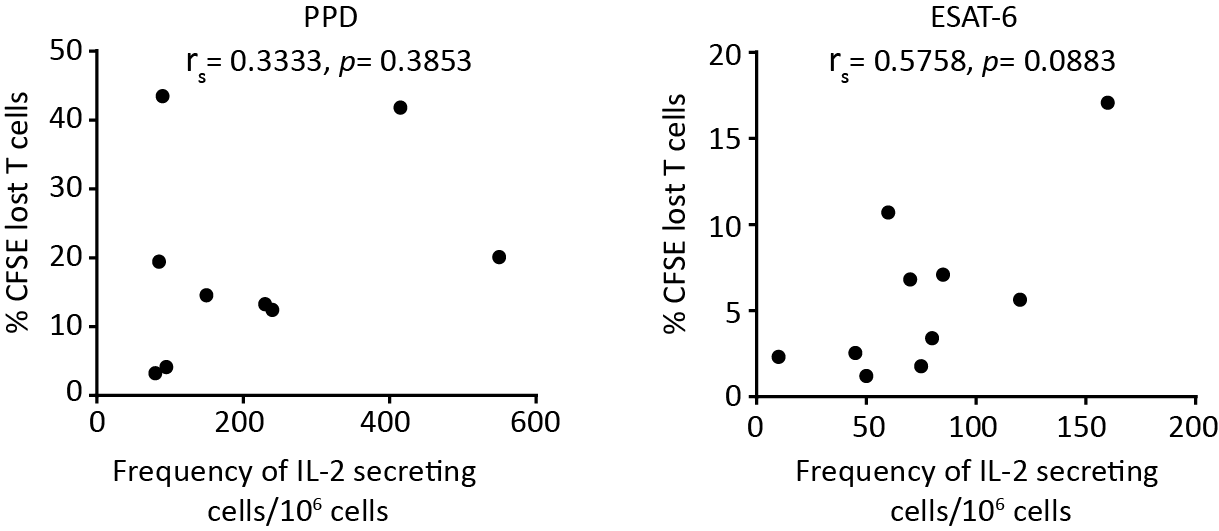

Supplement: Supplementary file 4 — Additional file 4: Figure S4. Association of IL-2 production and T cell proliferation in response to M. tuberculosis antigens. Correlation between frequency of IL-2-secreting cells and proliferating T cells among total PBMCs after stimulation with PPD (n = 9) and ESAT-6 (n = 10). Spearman correlation was used to calculate correlation coefficients (rs) and p values. [file 12865_2019_317_MOESM4_ESM.png]
